# Effect of true triaxial principal stress unloading rate on strain energy density of sandstone

**DOI:** 10.1038/s41598-024-66185-9

**Published:** 2024-07-02

**Authors:** Zhixi Liu, Guangming Zhao, Xiangrui Meng, Qingheng Gu

**Affiliations:** 1 School of Architectural Engineering, Tongling University, Tongling, 244061 China; 2 Anhui University of Science and Technology, State Key Laboratory of Mining Response and Disaster Prevention in Deep Coal Mines, Huainan, 232001 China

**Keywords:** Strain energy density, True triaxial compression, Unloading rate, True triaxial unloading principal stress experiments, Damage, Civil engineering, Solid Earth sciences

## Abstract

Deep rock are often in a true triaxial stress state. Studying the impacts of varying unloading speeds on their strain energy (SE) density is highly significant for predicting rock stability. Through true triaxial unloading principal stress experiments and true triaxial stress equilibrium unloading experiments on sandstone, this paper proposes a method to compute the SE density in a true triaxial compressive unloading principal stress test. This method aims to analyze the SE variation in rocks under the action of true triaxial unloading principal stresses. Acoustic emission is used to verify the correctness of the SE density calculation method in this paper. This study found that: (1) Unloading in one principal stress direction causes the SE density to rise in the other principal stress directions. This rise in SE, depending on its reversibility, can be categorized into elastic and dissipated SE. (2)When unloading principal stresses, the released elastic SE density in the unloading direction is influence by the stress path and rate. (3) The higher the unloading speed will leads to greater increases in the input SE density, elastic SE density, and dissipative SE density in the other principal stress directions. (4) The dissipated SE generated under true triaxial compression by unloading the principal stress is positively correlated with the damage to the rock; with an increase in unloading rate, there is a corresponding increase in the formation of cracks after unloading. (5) Utilizing the stress balance unloading test, we propose a calculation method for SE density in true triaxial unloading principal stress tests.

## Introduction

During the excavation of deep roadway, high-stress rock masses are damaged due to unloading from excavation, resulting in fracture and failure of surrounding rock masses. The true triaxial unloading principal stress test can effectively simulate these complex stress conditions, and thus reveal how rock gradually deforms during excavation and the accompanying energy evolution laws. By deeply understanding the mechanical behavior of deep rocks and their SE density laws, it provides a theoretical basis for better predicting and controlling the stability of surrounding rock in deep roadway.

During the excavation of deep roadway, rock will encounter unloading effect^1,2^. The unloading phenomenon refers to the gradual reduction of the originally high stress of the rock to a relatively lower stress level, sometimes even completely releasing all stress, entering a stress-free state^3,4^. In the research on the mechanical response of rocks under complex mechanical conditions, M.C. Richards et al. established a model to predict the mechanical unloading response of high-porosity sandstone. This model, which integrates experimentally observed changes in elastic volume and shear modulus with stress and plastic strain, has advanced theoretical research in rock mechanics^5^.When rocks are subjected to external actions, not only does SE accumulate, but SE dissipation also occurs. The instability of rock, fundamentally, is driven by a state transition caused by SE changes, with the variation in SE density during this process offering new insights into exploring the mechanism of rock failure. Deep rock are typically experience a true triaxial stress state, and studying the impacts of unloading rates on the SE of rocks can result in a more profound comprehension of the energy distribution and transformation before and after rock fracture, thus providing a scientific basis for rock failure prediction and the safety of deep mining.

Analyzing rock deformation and failure through thermodynamics to reveal the accumulation, dissipation, and release characteristics of SE has become a prominent topic in both domestic and international research^6–9^. Understanding SE density in rocks under complex stress conditions is essential for grasping the stress-strain behavior of rocks. Current research predominantly focuses on the evolution of SE density under uniaxial or conventional triaxial cyclic loading and unloading conditions^10–13^. These studies are often based on Hooke’s law, where rocks are assumed to behave as elastic bodies within certain stress limits, allowing for linear predictions of their behavior. However, recent investigations have highlighted that nonlinear characteristics significantly influence the actual behavior of rocks, particularly under unloading conditions^14–16^. Nonlinearities in rock unloading curves become evident when stress-strain paths diverge from the initial linear elastic response after stress removal. This behavior is particularly pronounced under cyclic loading-unloading scenarios, where the nonlinearity of unloading curves profoundly impacts the structural integrity and safety of rock engineering projects. Consequently, the accuracy of SE density calculation methods based on elastic theory is debatable. Recent advancements by Gong et al.^17^ introduced a method for SE density calculation using uniaxial single-cycle loading-unloading experiments on various rock types^18,19^. They discovered a linear energy storage and dissipation law in these tests, establishing the peak stress as a critical state. However, achieving loading-unloading at peak stress in uniaxial tests remains challenging, limiting the applicability of these findings to more complex stress states encountered in real-world engineering.

Despite significant progress in understanding rock SE density under uniaxial compression, there remains a substantial gap in knowledge regarding the behavior of SE density under true triaxial unloading conditions. These conditions are more representative of actual engineering environments, where the complexity and heterogeneity of rock structures play a crucial role. Specifically, the influence of unloading speed on SE density changes during triaxial unloading is a critical yet underexplored area of research. With the continuous development of research, some scholars have begun to explore the evolution of SE density in rock unloading under complex stress states. Research on rock unloading tests under complex stress conditions primarily focuses on conventional triaxial unloading tests. These can be categorized into axial stress unloading tests and confining pressure unloading experiments. Studies on axial stress unloading in conventional triaxial tests mainly revolve around SE and the evolution of mechanical characteristics in cyclic loading-unloading tests^20–23^. In conventional triaxial unloading confining pressure tests, many scholars have discovered that confining pressure significantly affects rock strength, elastic modulus, and mechanical properties^24–26^. Huang et al.^27^ investigated the accumulation, dissipation, and release mechanisms of SE under unloading confining pressure conditions. Li et al.^28^, Wang et al.^29^, and Su et al.^30^ simulated the stress paths for different rock types under mining stress conditions in their unloading confining pressure tests and studied their mechanical characteristics.

**Figure 1:**
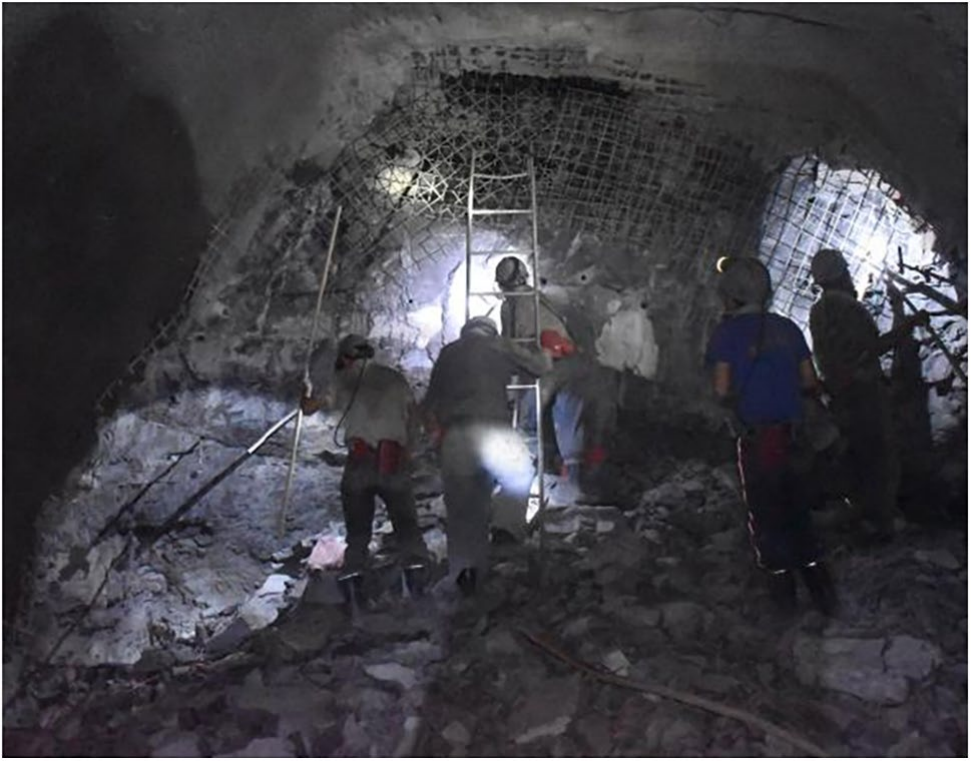
Schematic diagram of driving face in Luling Coal Mine.

Research on the deformation and failure process of rocks has been conducted for many years^31–35^. The initial stress state of the surrounding rock during unloading caused by excavation significantly affects its deformation and failure. Previous studies have investigated the effects under conventional triaxial unloading conditions^36^. For example, Li et al.^37^ found that lateral deformation is significant, and the volumetric deformation characteristics during unloading are comparable to the lateral deformation characteristics. Researchers have also analyzed the unloading process from an energy perspective, concluding that confining pressure significantly affects SE. They determined that the higher the confining pressure, the higher the ultimate energy storage capacity of the sample, making it more difficult for the sample to fail^38,39^. Understanding the evolution of SE density in rock unloading under complex stress conditions is important for predicting rock behavior during the unloading process and forecasting the deformation and failure of deep rocks. Particularly under true triaxial unloading conditions, the changes in SE density in other principal stress directions when one principal stress decreases, as well as the calculation methods for this issue, have not been systematically discussed. Furthermore, the specific impact of unloading rate on the SE density change process is an important research area, with theoretical significance for understanding rock response during unloading, predicting rock mass failure, and the stability of surrounding rock in deep roadways.Nevertheless, the research progress and theoretical framework in this field remain very limited. Fig. 1 shows the photos of surrounding rock support after excavation and unloading in Luling Coal Mine.

To address these knowledge gaps, this study proposes a series of systematic experimental designs and theoretical analyses to explore the change laws of sandstone SE density under true triaxial unloading circumstances. Through designed true triaxial main stress equilibrium unloading tests, this study systematically analyzes the influence of diverse unloading speeds on the change of sandstone SE density under true triaxial compressive states, and elucidates the mechanism by which unloading rate affects rock SE density. This provides a new perspective for analyzing the SE density of rock under complex stress conditions.

## Test methods

### Test system and sandstone

**Figure 2:**
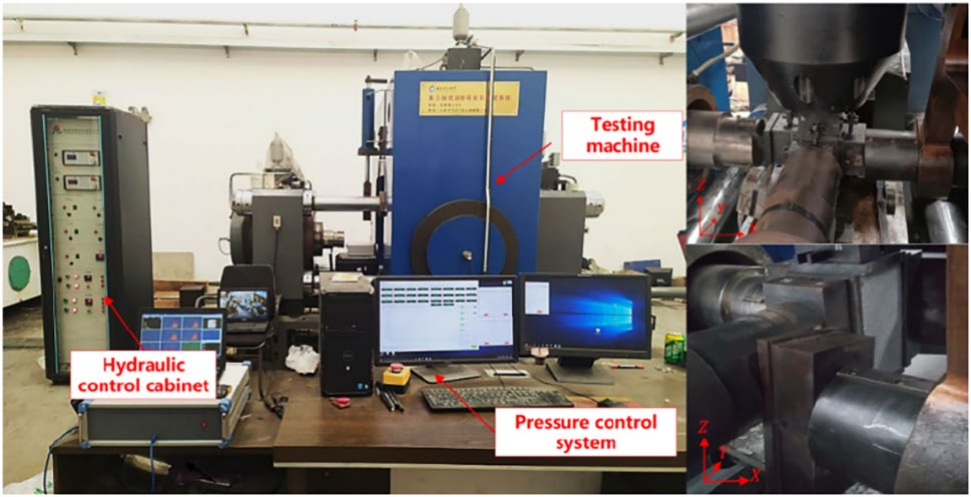
True triaxial rock mechanics test system.

To investigate the method of computing SE in rocks under the unloading of principal stress and the effect of unloading speed on SE density, main stress unloading tests were performed using a true triaxial test system (Fig. 2). The system employs 3 platen arrangement, where each of the three orthogonal axes is independently controlled to apply different principal stresses ($\sigma _{1}$, $\sigma _{2}$, $\sigma _{3}$) on the sandstone samples.The loading system is equipped with high-precision load cells and displacement transducers to accurately measure the applied forces and deformations. To minimize friction effects between the platens and the sample, we applied white Vaseline to the sample. White Vaseline significantly reduce frictional resistance, ensuring uniform stress distribution.

**Figure 3:**
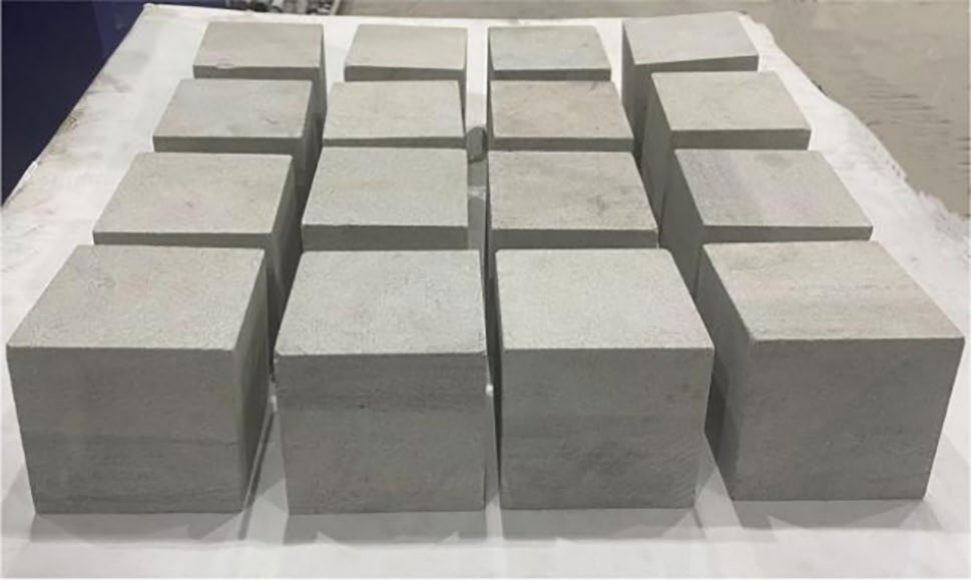
Sandstone samples.

The sandstone used in this study originates from a single source, and the specimens were processed into cubes with a side length of 100 mm. The uniaxial compressive strength of the sandstone is approximately 45.2MPa. Figure 3 displays the sandstone samples used in this study.

Petrographic Analysis: A detailed petrographic analysis of the sandstone samples has been conducted to characterize their mineralogical composition and textural properties. Below are the key findings: Mineral Composition: The sandstone is predominantly composed of quartz (approximately 85%), feldspar (about 10%), and minor amounts of mica and lithic fragments (around 5%). Grain Size and Sorting: The grains are generally well-sorted, with an average grain size ranging from 0.2 mm to 0.5 mm. The texture is medium-grained, indicative of a relatively high degree of uniformity in sedimentation. Cementation: The sandstone exhibits silica cementation, with some areas showing partial clay cement. The overall porosity of the samples is estimated to be around 12%, based on both petrographic observations and laboratory porosity measurements. The sandstone samples used in this study were obtained from the Pz (Paleozoic erathem) Formation, located in Luling Coal mine in Anhui province.

### Test procedure

The test procedure for minimum principal stress ($\sigma _{3}$) - intermediate principal stress ($\sigma _{2}$) - maximum principal stress ($\sigma _{1}$) is as follow.

Step 1: The loading rates for the $\sigma _{3}$, $\sigma _{2}$, and $\sigma _{1}$directions are 0.1, 0.15, and 0.2MPa/s, respectively, until reaching the levels of 20MPa, 30MPa, and 40MPa.

**Figure 4:**
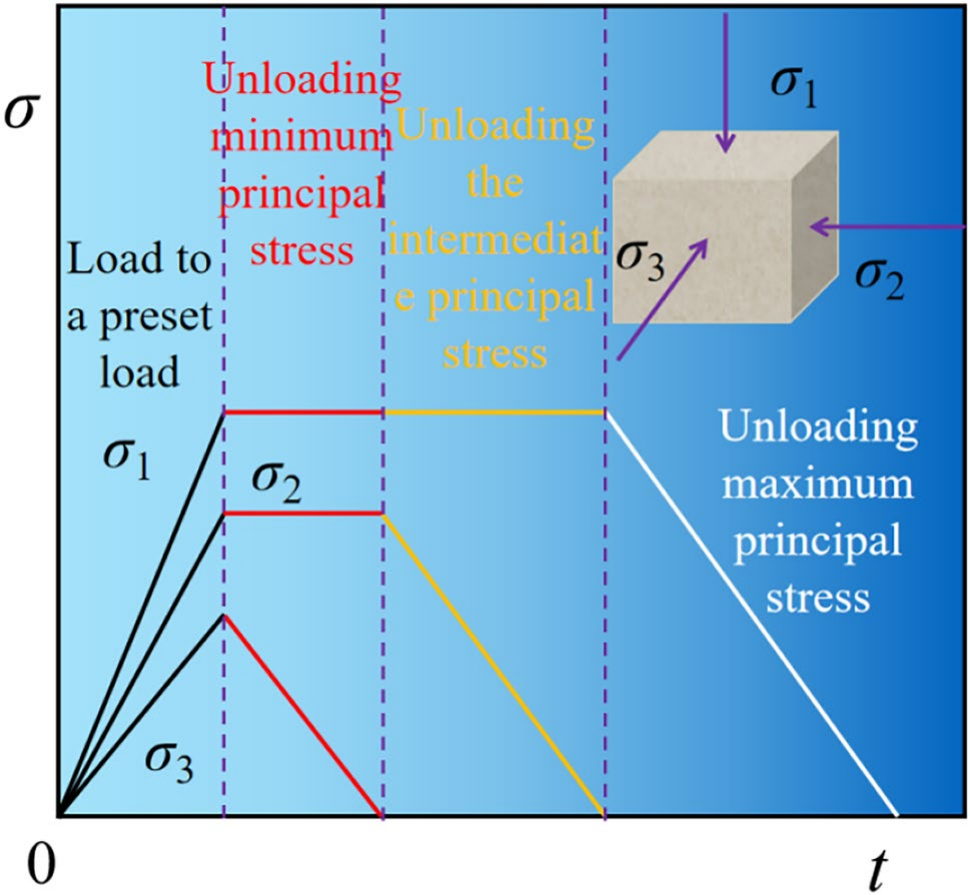
Stress path diagram of $\sigma _{3} -\sigma _{2} -\sigma _{1}$.

Step 2: Unloading involves five different rates of 0.1, 0.5, 1.0, 1.5, and 2.0MPa/s. The specific unloading procedure is as follows: Firstly,unload $\sigma _{3}$ to 0, then unload the $\sigma _{2}$ to 0, and finally, unload the $\sigma _{1}$ to 0. The unloading schematic is depicted in Fig. 4. The $\sigma _{3}$-$\sigma _{2}$-$\sigma _{1}$ tests conducted in this paper are shown in Table 1.

**Table 1:** $\sigma _{3}- \sigma _{2} -\sigma _{1}$ experiment unloading rate.(MPa/s).

Sample	$\sigma _{3}$	$\sigma _{2}$	$\sigma _{1}$
$$S_{1} $$	0.1	0.1	0.1
$$S_{2} $$	0.5	0.5	0.5
$$S_{3} $$	1.0	1.0	1.0
$$S_{4} $$	1.5	1.5	1.5
$$S_{5} $$	2.0	2.0	2.0

### Test curve analysis

**Figure 5:**
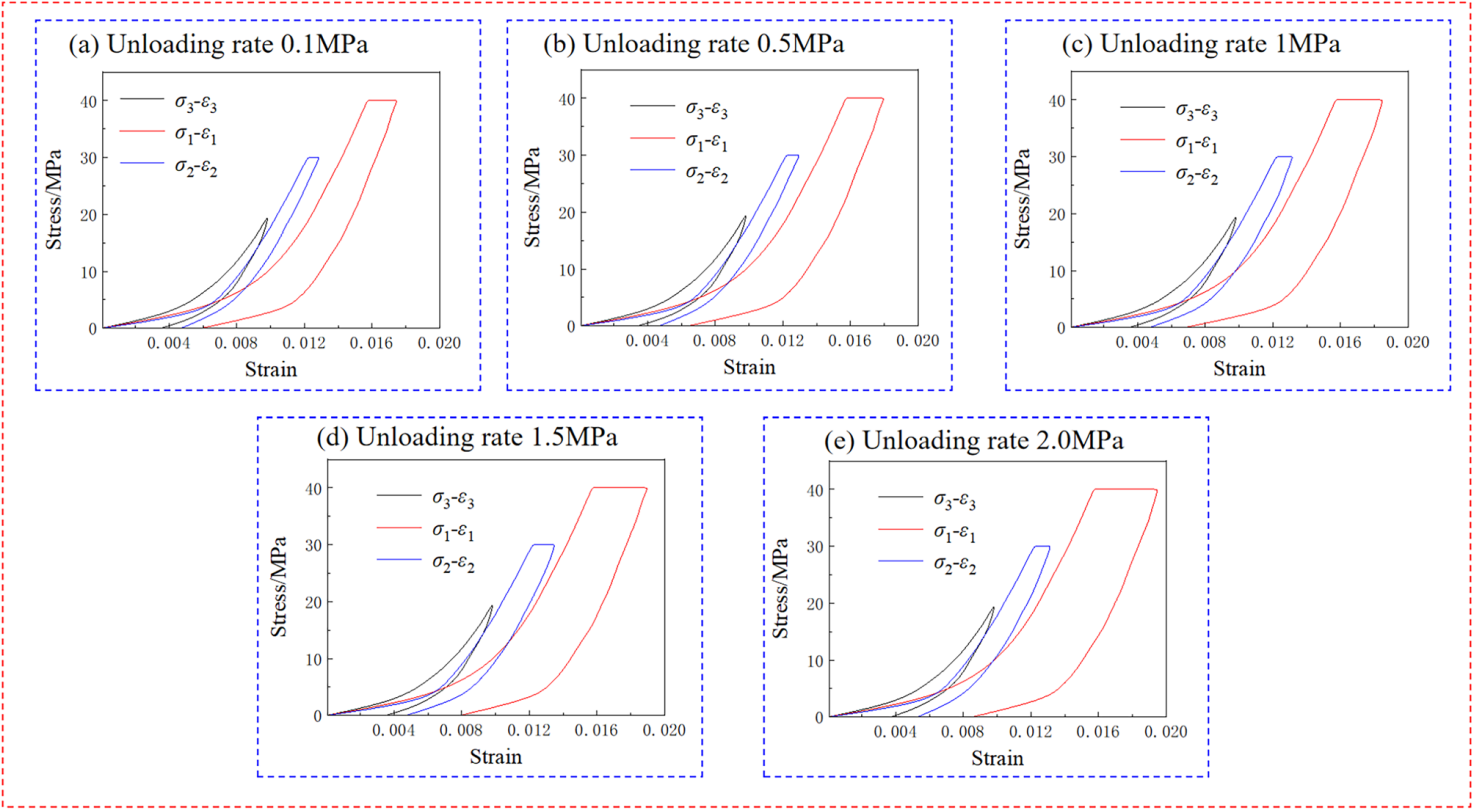
Stress-strain curve of $\sigma _{3} -\sigma _{2} -\sigma _{1}$ test.

Figure 5 shows the stress-strain curves of rock under diverse unloading speeds. During the $\sigma _{3} -\sigma _{2} -\sigma _{1}$ experiment, unloading $\sigma _{3}$ will result in an raising in strain in the $\sigma _{2}$ and $\sigma _{1}$ directions.According to the generalized Hooke’s law, unloading stress in one principal stress direction usually results in an increase in elastic deformation in $\sigma _{2}$ and $\sigma _{1}$, as the material’s Poisson effect causes a corresponding change in dimensions perpendicular to the unloaded direction. However, rocks are not purely elastic materials, and their deformation and failure processes often exhibit significant nonlinear characteristics. During the unloading $\sigma _{3}$, the stress distribution inside the sandstone adjusts, often leading to stress concentration phenomena in rock, resulting in residual strain in the $\sigma _{2}$ and$\sigma _{1}$ directions. After unloading $\sigma _{3}$, if further unloading of $\sigma _{2}$ is carried out, an increase in strain in the $\sigma _{2}$ direction will be observed.

### True triaxial stress equilibrium unloading test

In the aforementioned study on the true triaxial unloading principal stress test, it was observed that under complex stress conditions, unloading one principal stress direction in a true triaxial test induces changes in strain in all three principal stress directions of the rock, thus complicating the differentiation of strain and SE density analysis. Under true triaxial compression conditions, if the three principal stress directions ($\sigma _{1}$, $\sigma _{2}$, $\sigma _{3}$) are proportionally unloaded to zero simultaneously, it indicates that the rock transitions from a compressed state to a stress-free state. Generally, if the unloading process is uniform and gradual, and the pre-unloading stress state has not surpassed the rock’s elastic limit, the rock theoretically will not sustain damage. The reason for this is that during proportional unloading, the stress in all three principal stress directions decreases simultaneously, helping to prevent internal damage due to stress redistribution or stress imbalances. To analyze the true triaxial unloading principal stress test presented in this paper, two sets of true triaxial stress equilibrium unloading tests were designed.

This study conducted a series of supplementary experiments to analyze the true triaxial unloading principal stress test. This test can be understood in four stages: first, loading to the preset load; second, unloading the $\sigma _{3}$; third, unloading the $\sigma _{2}$; and fourth, unloading the $\sigma _{1}$.

**Figure 6:**
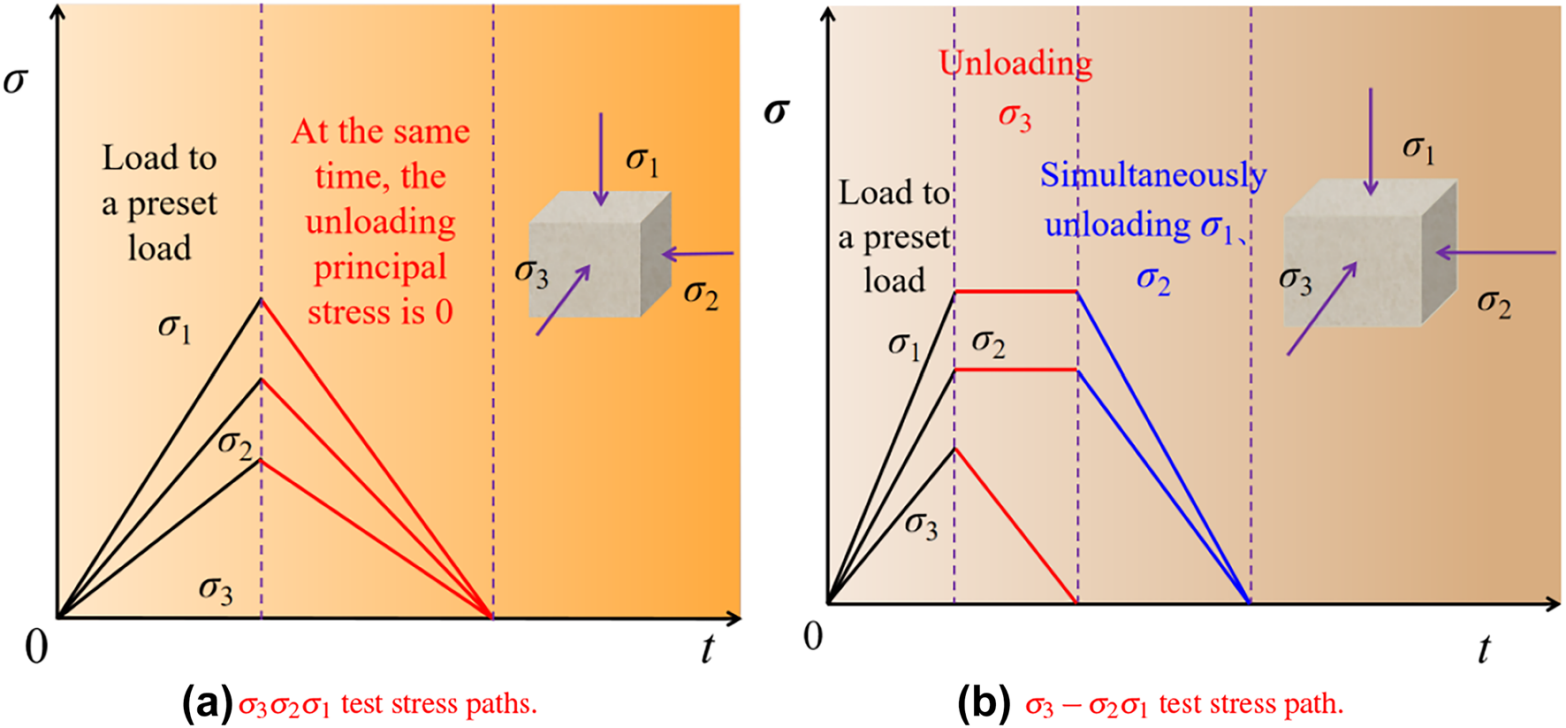
Test stress path.

The first approach involves simultaneous triaxial unloading, where the $\sigma _{3}$, $\sigma _{2}$, $\sigma _{1}$ are loaded to the initial stress state at the same time, then unloaded simultaneously, ensuring that all three principal stresses are reduced to zero simultaneously. For ease of analysis, this experiment is referred to as: the $\sigma _{3}$ $\sigma _{2}$ $\sigma _{1}$ test, intended to understand the elastic and plastic strain in the three principal stress directions when loaded to the preset load during true triaxial compression. The stress path is illustrated in Fig. 6a. The second approach involves unloading $\sigma _{3}$ first, followed by simultaneous unloading of $\sigma _{2}$ and $\sigma _{1}$, while ensuring that $\sigma _{2}$ and $\sigma _{1}$ directions are simultaneously unloaded to zero. For ease of description, this experimental approach is briefly referred to as: the $\sigma _{3}$-$\sigma _{2}$ $\sigma _{1}$ test, which can analyze the second stage. Through the two aforementioned true triaxial stress equilibrium unloading tests, the strain in the first and second stages of the true triaxial unloading principal stress test can be understood. In the third stage, only the intermediate and $\sigma _{1}$ remain. The effect of unloading the $\sigma _{2}$ on the strain and SE density in the $\sigma _{1}$ direction can be understood through the fourth step of unloading the $\sigma _{1}$. Therefore, no additional auxiliary tests are set. The schematic of the stress path is shown in Fig. 6b.

The specific implementation steps of the $\sigma _{3} \sigma _{2} \sigma _{1}$ test are:

Step 1: Same as step 1 of the $\sigma _{3} -\sigma _{2} -\sigma _{1}$ test.

Step 2: The values of $\sigma _{3}$, $\sigma _{2}$, and $\sigma _{1}$ are 0.1MPa/s, 0.15MPa/s, and 0.2MPa/s, respectively. To achieve consider the influence of unloading rate on strain evolution, five rates were set in the $\sigma _{3} \sigma _{2} \sigma _{1}$ experiment, as demonstrated in Table 2.

**Table 2:** $\sigma _{3} \sigma _{2} \sigma _{1}$ experiment unloading rate.(MPa/s).

Sample	$\sigma _{3}$	$\sigma _{2}$	$\sigma _{1}$
$$Z_{1} $$	0.1	0.15	0.2
$$Z_{2} $$	0.5	0.75	1
$$Z_{3} $$	1.0	1.5	2
$$Z_{4} $$	1.5	2.25	3
$$Z_{5} $$	2.0	3.0	4

The specific steps of the $\sigma _{3}$-$\sigma _{2}$ $\sigma _{1}$ test are:

Step 1: Same as step 1 of the $\sigma _{3} -\sigma _{2} -\sigma _{1}$ test.

Step 2: Unload $\sigma _{3}$ in the direction of 0. The test of $\sigma _{3}$-$\sigma _{2}$ $\sigma _{1}$ can unload $\sigma _{3}$ at five rates: 0.1, 0.5, 1, 1.5, and 2 MPa /s.

Step 3: Simultaneously unload the $\sigma _{2}$ and $\sigma _{1}$. The principal stress in the $\sigma _{2}$ direction is unloaded at 0.15MPa/s, and the principal stress in the $\sigma _{1}$ direction is 0.2MPa/s. This unloading rate can ensure that $\sigma _{1}$ and $\sigma _{2}$ are simultaneously unloaded at 0. As demonstrated in Table 3.

**Table 3:** $\sigma _{3} -\sigma _{2} \sigma _{1}$ experiment unloading rate.(MPa/s).

Sample	$\sigma _{3}$	$\sigma _{2}$	$\sigma _{1}$
$$Y_{1} $$	0.1	0.15	0.2
$$Y_{2} $$	0.5	0.15	0.2
$$Y_{3} $$	1.0	0.15	0.2
$$Y_{4} $$	1.5	0.15	0.2
$$Y_{5} $$	2.0	0.15	0.2

## Calculation method of strain and SE density for unloading principal stress test

Unloading a principal stress direction will lead to a gradual decrease in strain in that direction and cause changes in strain in the $\sigma _{2}$ and$\sigma _{1}$. The specific changes in the actual process need to be analyzed through supplementary experiments.

### $\sigma _{3} -\sigma _{2} -\sigma _{1}$ test strain analysis for unloading $\sigma _{3}$

**Figure 7:**
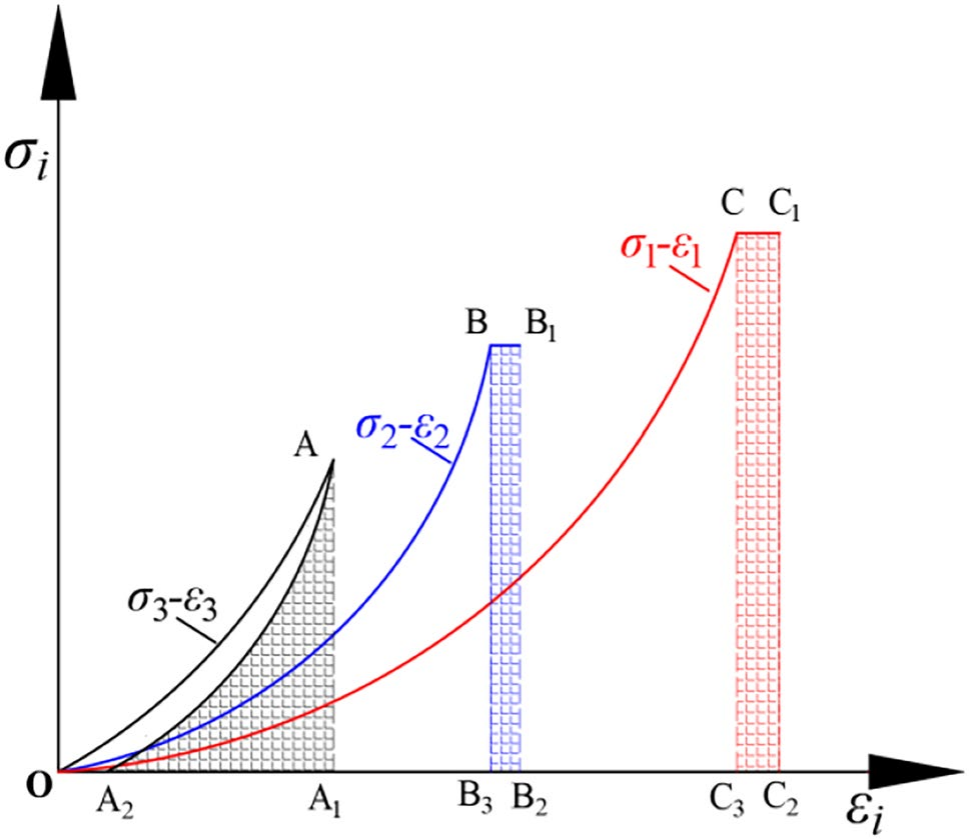
$\sigma _{3}$ unloading, the alterations in SE in three orientations..

After unloading $\sigma _{3}$, the alterations in SE in three orientations of the rock are shown in Fig. 7. In rock mechanics, it is crucial to assess and understand the evolution of SE density in sandstone under different stress conditions. Uniaxial compression tests involvely stress acting in a single direction, whereas true triaxial unloading tests are significantly more complex. In uniaxial compression experiments, the SE density of rocks can generally be described relatively simply specific rock mechanics models or equations. However, in a true triaxial state, after the principal stress is unloaded, the evolution of SE density in three directions often cannot be fully represented by a single formula. This is because the rock’s response is affected by stress conditions in all three orthogonal directions. Figure 7 illustrates the alterations in SE density in $\sigma _{3}$, $\sigma _{2}$ and $\sigma _{1}$ of the sandstone under the action of unloading $\sigma _{3}$.

For the purpose of analysis, the displacements at various points in Fig. 7 are replaced by letters. In Fig. 7, the strain at point *A* is denoted as $$\varepsilon _{x_{1} } $$, the strain at point $$A_{2} $$ as $$\varepsilon _{x_{2} } $$, the strain at point *B* as $$\varepsilon _{y_{1} } $$, the strain at point $$B_{1} $$ as $$\varepsilon _{y_{2} } $$, the strain at point *C* as $$\varepsilon _{z_{1} } $$, and the strain at point $$C_{1} $$ as $$\varepsilon _{z_{2} } $$.

The elastic strain after $\sigma _{3}$ unloading is:

1 $$\begin{aligned} \varepsilon _{x_{e} }^{\prime }=\varepsilon _{x_{1} }-\varepsilon _{x_{2} } \end{aligned}$$

The increase of strain in the $\sigma _{2}$ direction after $\sigma _{3}$ unloading is as follows:

2 $$\begin{aligned} \bigtriangleup \varepsilon ^{'} _{y} =\varepsilon _{y_{2} } -\varepsilon _{y_{1} } \end{aligned}$$

After unloading $\sigma _{3}$, the increase of strain in the direction of $\sigma _{1}$ is as follows:

3 $$\begin{aligned} \bigtriangleup \varepsilon ^{'} _{z} =\varepsilon _{z_{2} } -\varepsilon _{z_{1} } \end{aligned}$$

After the unloading of $\sigma _{3}$, two main issues require further discussion and analysis. Firstly, we need to determine whether unloading $\sigma _{3}$ significantly impacts the strain evolution in that direction. Secondly, unloading $\sigma _{3}$ may induce increased of strain in the directions of $\sigma _{2}$ and $\sigma _{1}$, which is equally important. Moreover, we need to quantify the specific values of this increased strain. This means identifying the types of increased strain and determining their specific values through precise measurement and calculation. These data are crucial for understanding the changes in mechanical behavior during the unloading process and for guiding future engineering and scientific research.

### Ancillary tests for $\sigma _{3} -\sigma _{2} -\sigma _{1}$ test analysis

**Figure 8:**
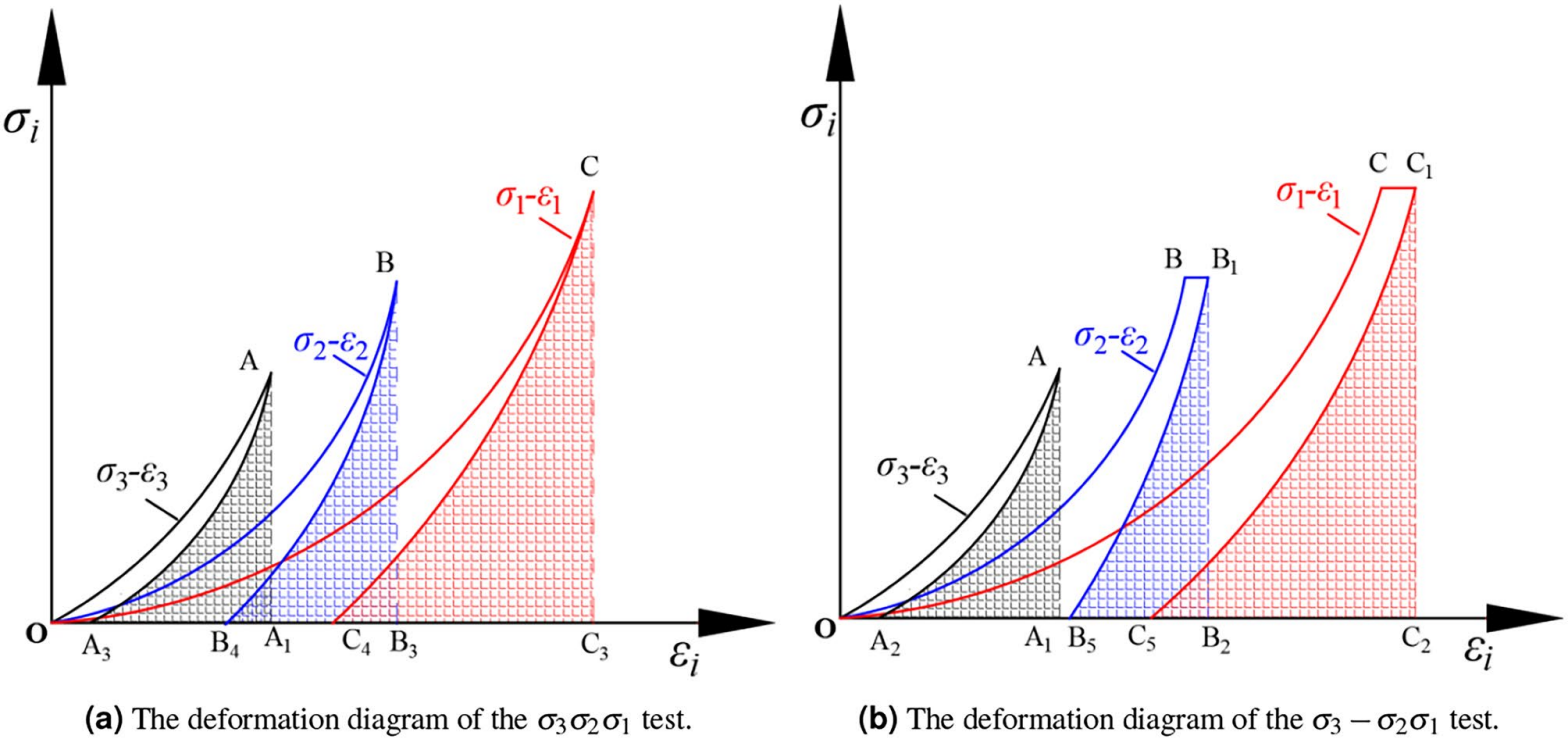
The deformation diagram.

Figure 8a shows the stress-strain schematic for the $\sigma _{3} \sigma _{2} \sigma _{1}$ test, intended to understand the elastic and plastic strain in the three principal stress directions when loaded to the preset load during true triaxial compression. Figure 8b illustrates the stress-strain curve for the $\sigma _{3} -\sigma _{2} \sigma _{1}$ test, which can analyze the second stage. This involves the strains in the intermediate and $\sigma _{1}$ directions after the $\sigma _{3}$ is unloaded to zero. This clarifies the rationale behind conducting both $\sigma _{3} \sigma _{2} \sigma _{1}$ and $\sigma _{3} -\sigma _{2} \sigma _{1}$ tests in the manuscript. The stress-strain diagram of the $\sigma _{3} \sigma _{2} \sigma _{1}$ test is drawn, as Fig. 8a. The stress-strain diagram of the $\sigma _{3} -\sigma _{2} \sigma _{1}$ test is shown in Fig. 8b.

To facilitate analysis, the strain at point $B_{4}$ in Fig. 8a is denoted as $\varepsilon _{y_{3} }$, and the strain at point $B_{5}$ in Fig. 8b is denoted as $\varepsilon _{y_{4} }$. The strain at point $C_{4}$ in Fig. 8a is denoted as $\varepsilon _{z_{3} }$, and the strain at point $C_{5}$ in Fig. 8b is denoted as $\varepsilon _{z_{4} }$. By comparing the residual and elastic strain in the $\sigma _{3}$ direction between the $\sigma _{3} \sigma _{2} \sigma _{1}$ experiment and the $\sigma _{3} -\sigma _{2} -\sigma _{1}$ experiment, we can understand the influence of the unloading stress path on the strain in the $\sigma _{3}$ direction. After unloading $\sigma _{3}$, the increase in strain in the $\sigma _{2}$ direction, consisting of elastic strain and residual strain, is as follows:

4 $$\begin{aligned} \left\{ \begin{array}{l} \Delta \varepsilon _{y _{e} }^{\prime }=\left( \varepsilon _{y _{2}}-\varepsilon _{y _{4}}\right) -\left( \varepsilon _{y _{1}}-\varepsilon _{y _{3}}\right) \\ \Delta \varepsilon _{y _{r}}^{\prime }=\Delta \varepsilon _{y}^{\prime }-\Delta \varepsilon _{y _{e}}^{\prime } \end{array}\right. \end{aligned}$$

Where $\Delta \varepsilon _{y _{e} }^{\prime }$ is the increased elastic strain in $\sigma _{2}$ after unloading $\sigma _{3}$, and $\Delta \varepsilon _{y _{r}}^{\prime }$ is the increased residual strain in the direction of $\sigma _{2}$ after unloading $\sigma _{3}$.

After unloading $\sigma _{3}$, the elastic and residual strain in the increased strain in the $\sigma _{1}$ direction are:

5 $$\begin{aligned} \left\{ \begin{array}{l} \Delta \varepsilon _{z _{e} }^{\prime }=\left( \varepsilon _{z _{2}}-\varepsilon _{z _{4}}\right) -\left( \varepsilon _{z _{1}}-\varepsilon _{z _{3}}\right) \\ \Delta \varepsilon _{z _{r}}^{\prime }=\Delta \varepsilon _{z}^{\prime }-\Delta \varepsilon _{z _{e}}^{\prime } \end{array}\right. \end{aligned}$$

Where $\Delta \varepsilon _{z _{e} }^{\prime }$ is the increased elastic strain of $\sigma _{1}$ after unloading $\sigma _{3}$, and $\Delta \varepsilon _{z _{r}}^{\prime }$ is the increased residual strain in the direction of $\sigma _{1}$ after unloading $\sigma _{3}$.

**Figure 9:**
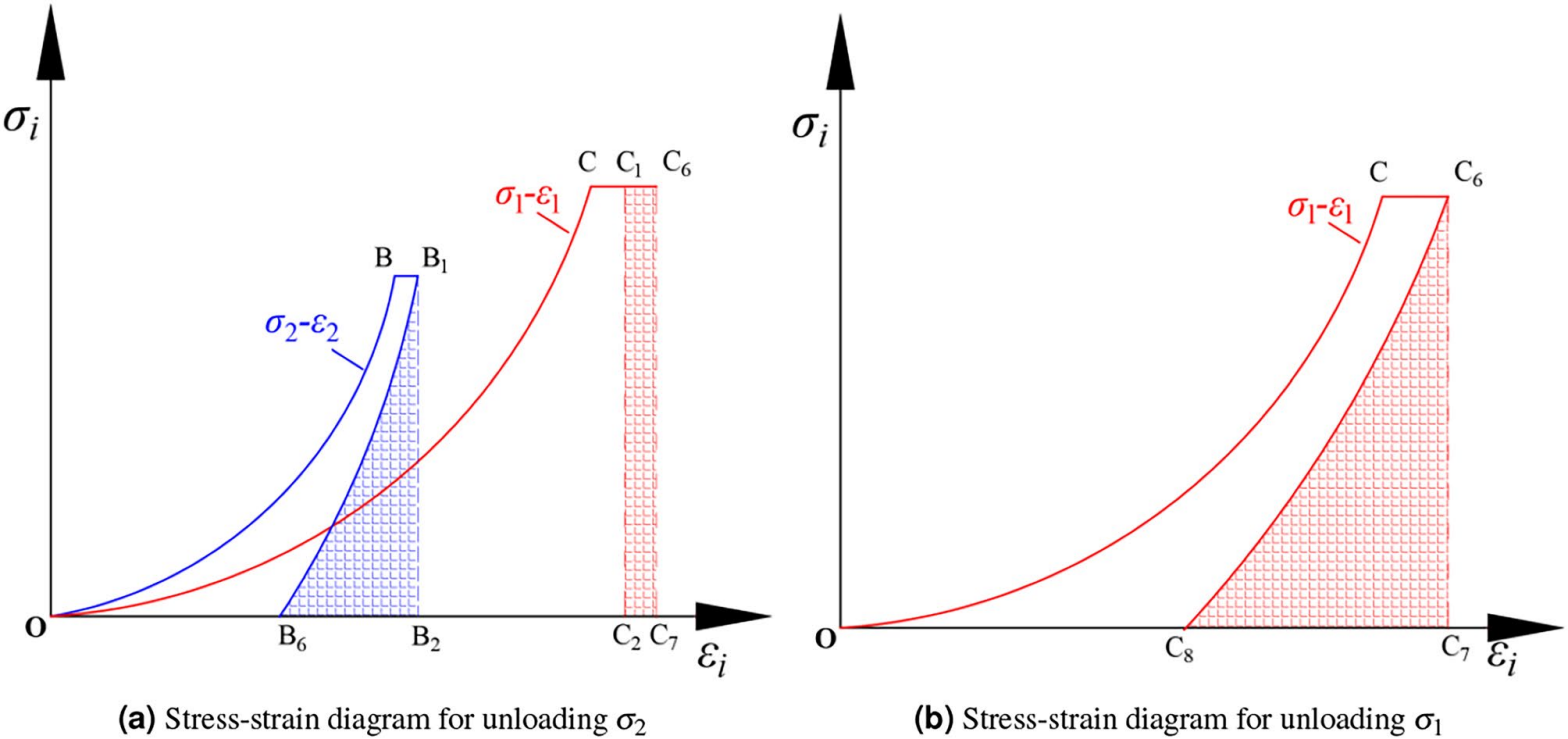
The stress-strain diagrams.

Fig. 9a represents the $\sigma _{2}$ unloading phase of the $\sigma _{3} -\sigma _{2} -\sigma _{1}$ test, the third stage. After this stage, only the $\sigma _{1}$ remains, entering the fourth stage, which is considered uniaxial unloading. Elastic and residual strains in the $\sigma _{1}$ direction are obtained through this unloading. The strains and SE density in the true triaxial unloading principal stress test can be analyzed using both $\sigma _{3} -\sigma _{2} -\sigma _{1}$ and $\sigma _{3} -\sigma _{2} \sigma _{1}$ tests. During the $\sigma _{3} -\sigma _{2} -\sigma _{1}$ experiment, after unloading $\sigma _{3}$ to zero, we will continue to unload $\sigma _{2}$. At this stage, the process of unloading $\sigma _{2}$ also faces two significant issues require in-depth discussion and analysis: Firstly, what impact does the stress path have on the strain in the $\sigma _{2}$ direction; secondly, what are the changes and effects on the strain in the $\sigma _{1}$ direction when $\sigma _{2}$ is further unloaded. In the experiment, we first reduce $\sigma _{3}$ to zero and then proceed to unload $\sigma _{2}$, the stress-strain diagram of which is shown in Fig. 9a. After that, we continue to unload $\sigma _{1}$ from the state where $\sigma _{2}$ has been reduced to zero, as demonstrated in Fig. 9b.

In order to facilitate analysis, the strain of point $B_{6}$ in the strain diagram in Fig. 9a is $\varepsilon _{y_{5} }$, and that of point $C_{6}$ is $\varepsilon _{z_{5} }$. The displacement of $C_{8}$ in Fig.9b is $\varepsilon _{z_{6} }$. When the principal stress in *y* direction is unloaded to 0, the strain in *z* direction increases, which can be described by the formula as follows:

6 $$\begin{aligned} \bigtriangleup \varepsilon ^{\prime \prime } _{z } =\varepsilon _{z_{5} } -\varepsilon _{z_{2} } \end{aligned}$$

Formula $\bigtriangleup \varepsilon ^{\prime \prime } _{z }$ represents the strain increased in the $\sigma _{1}$ direction after unloading $\sigma _{2}$.

The elastic and residual strain in the increasing strain in the $\sigma _{1}$ direction can be described as:

7 $$\begin{aligned} \left\{ \begin{array}{l} \Delta \varepsilon _{z _{e} }^{\prime \prime }=\left( \varepsilon _{z _{5}}-\varepsilon _{z _{6}}\right) -\left( \varepsilon _{z _{2}}-\varepsilon _{z _{4}}\right) \\ \Delta \varepsilon _{z _{r}}^{\prime \prime }=\Delta \varepsilon _{z}^{\prime \prime }-\Delta \varepsilon _{z _{e}}^{\prime \prime } \end{array}\right. \end{aligned}$$

Where $\Delta \varepsilon _{z _{e} }^{\prime \prime }$ and $\Delta \varepsilon _{z _{r}}^{\prime \prime }$ represent the increased elastic and residual strain of $\sigma _{1}$ direction after loading $\sigma _{2}$.

## Calculation method of SE density for $\sigma _{3} -\sigma _{2} -\sigma _{1}$ test

Based on the method for calculating strain and its analysis in the $\sigma _{3} -\sigma _{2} -\sigma _{1}$ experiment, the input SE density under the initial stress state can be derived by integrating the stress-strain curve. The input energy density generated by true triaxial compression under the initial stress state is as follows:

8 $$\begin{aligned} u=\int \limits _{0}^{\varepsilon _{x_{1} } } \sigma _{x} d\varepsilon _{x} +\int \limits _{0}^{\varepsilon _{y_{1} } } \sigma _{y} d\varepsilon _{y}+\int \limits _{0}^{\varepsilon _{z_{1} } } \sigma _{z} d\varepsilon _{z} \end{aligned}$$

Where: *u* is the input SE density under the initial stress state, the unit is $mJ\cdot mm^{-3}$; $\sigma _{3}$, $\sigma _{2}$ and $\sigma _{1}$ are the stresses in the *x*, *y* and *z* directions of the true triaxial testing machine; $\varepsilon _{x}$, $\varepsilon _{y}$, and $\varepsilon _{z}$ are strains in the true $\sigma _{3}$, $\sigma _{2}$ and $\sigma _{1}$ directions. Unloading $\sigma _{3}$ releases the elastic energy in the $\sigma _{3}$ direction:

9 $$\begin{aligned} u^{x} _{e} =\int \limits _{x_{1} }^{\varepsilon _{x_{2} } } \sigma _{x} d\varepsilon _{x} \end{aligned}$$

Where: $u^{x} _{e}$ indicates the elastic SE released by the rock mass when $\sigma _{3}$ is unloaded.

When $\sigma _{3}$ is unloaded, the resulting input SE density in the $\sigma _{2}$ and $\sigma _{1}$ is:

10 $$\begin{aligned} {\left\{ \begin{array}{ll}\bigtriangleup u_{y _{1}} =\int \limits _{y_{1} }^{\varepsilon _{y_{2} } } \sigma _{y} d\varepsilon _{y}\\ \bigtriangleup u_{z _{1}} =\int \limits _{z_{1} }^{\varepsilon _{z_{2} } } \sigma _{z} d\varepsilon _{z} \end{array}\right. } \end{aligned}$$

Where, $\bigtriangleup u_{y _{1}}$ and $\bigtriangleup u_{z _{1}}$ represent the SE density in $\sigma _{2}$ and $\sigma _{1}$ directions respectively after unloading $\sigma _{3}$.

The areas enclosed by points $AA_{1}A_{3}$, $BB_{3}B_{4}$, and $CC_{3}C_{4}$ Fig. 8a represent the elastic SE stored in the $\sigma _{3}$, $\sigma _{2}$, and $\sigma _{1}$ directions under the initial stress level.

11 $$\begin{aligned} {\left\{ \begin{array}{ll} u^{e} _{x} =\int \limits _{\varepsilon _{x_{3} } }^{\varepsilon _{x_{1} } } \sigma _{x} d\varepsilon _{x} \\ u^{e} _{y} =\int \limits _{\varepsilon _{y_{3} } }^{\varepsilon _{y_{1} } } \sigma _{y} d\varepsilon _{y} \\ u^{e} _{z} =\int \limits _{\varepsilon _{z_{3} } }^{\varepsilon _{z_{1} } } \sigma _{z} d\varepsilon _{z} \end{array}\right. } \end{aligned}$$

Where $u^{e} _{x}$, $u^{e} _{y}$ and $u^{e} _{z}$ represent the elastic SE density stored by the rock in $\sigma _{3}$, $\sigma _{2}$, and $\sigma _{1}$ directions at the initial stress level.

After unloading $\sigma _{3}$, the elastic SE stored of $\sigma _{2}$ and $\sigma _{1}$ can be described as:

12 $$\begin{aligned} {\left\{ \begin{array}{ll} u^{y} _{e} =\int \limits _{\varepsilon _{y_{4} } }^{\varepsilon _{y_{2} } } \sigma _{y} d\varepsilon _{y} \\ u^{z} _{e} =\int \limits _{\varepsilon _{z_{4} } }^{\varepsilon _{z_{2} } } \sigma _{z} d\varepsilon _{z} \end{array}\right. } \end{aligned}$$

Where $u^{y} _{e}$ and $u^{z} _{e}$ represent the elastic SE density stored in $\sigma _{2}$ and $\sigma _{1}$ directions after unloading the stress in $\sigma _{3}$ direction.

After unloading in the *x* direction, the three principal stress direction changes can be described as:

13 $$\begin{aligned} {\left\{ \begin{array}{ll}\bigtriangleup u^{x} _{e} =u^{e }_{x} -u^{x}_{e} \\ \bigtriangleup u^{y} _{e} =u^{e }_{y} -u^{y}_{e} \\ \bigtriangleup u^{z} _{e} =u^{e }_{z} -u^{z}_{e} \end{array}\right. } \end{aligned}$$

$\bigtriangleup u^{x} _{e}$, $\bigtriangleup u^{y} _{e}$ and $\bigtriangleup u^{z} _{e}$ are $\sigma _{3} -\sigma _{2} -\sigma _{1}$ test when unloading in the $\sigma _{3}$ direction, and the change value of the elastic SE density in the direction of $\sigma _{3}$, $\sigma _{2}$ and $\sigma _{1}$.

After unloading $\sigma _{3}$ to 0 and then unloading $\sigma _{2}$, the elastic SE released in the $\sigma _{2}$ direction can be expressed as:

14 $$\begin{aligned} u^{y^{\prime } } _{e} =\int \limits _{\varepsilon _{y_{4} } }^{\varepsilon _{y_{3} } } \sigma _{y} d\varepsilon _{y} \end{aligned}$$

Where: $u^{y^{\prime } } _{e}$ represents the elastic SE density released by $\sigma _{2}$ after $\sigma _{3}$ is unloaded.

After unloading $\sigma _{3}$ and continuing unloading $\sigma _{2}$, the input SE density in the $\sigma _{1}$ direction due to unloading $\sigma _{2}$ is:

15 $$\begin{aligned} \bigtriangleup u_{z_{2} } =\int \limits _{\varepsilon _{z_{6} } }^{\varepsilon _{z_{2} } } \sigma _{z}d\varepsilon _{z} \end{aligned}$$

Where $\bigtriangleup u_{z_{2} }$ represents the input SE density in the $\sigma _{1}$ direction after unloading $\sigma _{2}$.

When $\sigma _{2}$ is 0, the elastic energy released when $\sigma _{1}$ is unloaded is as follows:

16 $$\begin{aligned} u^{z^{\prime } }_{e} = \int \limits _{\varepsilon _{z_{6} } }^{\varepsilon _{z_{5} } } \sigma _{z} d\varepsilon _{z} \end{aligned}$$

Where $u^{z^{\prime } }_{e}$ indicates the elastic energy released when $\sigma _{1}$ is unloaded.

After unloading $\sigma _{2}$ to 0, increases the elastic SE density of the $\sigma _{1}$ direction as follows:

17 $$\begin{aligned} \bigtriangleup u^{z^{\prime } }_{e} = u^{z^{\prime } }_{e} -u^{z }_{e} \end{aligned}$$

Where $\bigtriangleup u^{z^{\prime } }_{e}$ is the increase of elastic SE density in the $\sigma _{1}$ direction after unloading $\sigma _{2}$.

## Verification of SE density calculation method

In the study of damage evolution in solid materials, many scholars believe that acoustic emission (AE) ring-down counts reflect material damage, and when new cracks initiate and propagate inside the material, AE ring-down counts activity is strong^40–42^. In the study of uniaxial cyclic loading and unloading of rock, unloading does not cause rock damage; it is a process of elastic SE release, and the area enclosed by the unloading stress-strain curve and the strain axis represents the density of elastic SE stored under uniaxial compression. AE ring-down counts of rock enter an “intermittent period” during the unloading stage of uniaxial cyclic loading and unloading tests^43^. Based on the above research results, it can be considered that AE ring-down counts activity in rock is nearly absent when there is no damage.

**Figure 10:**
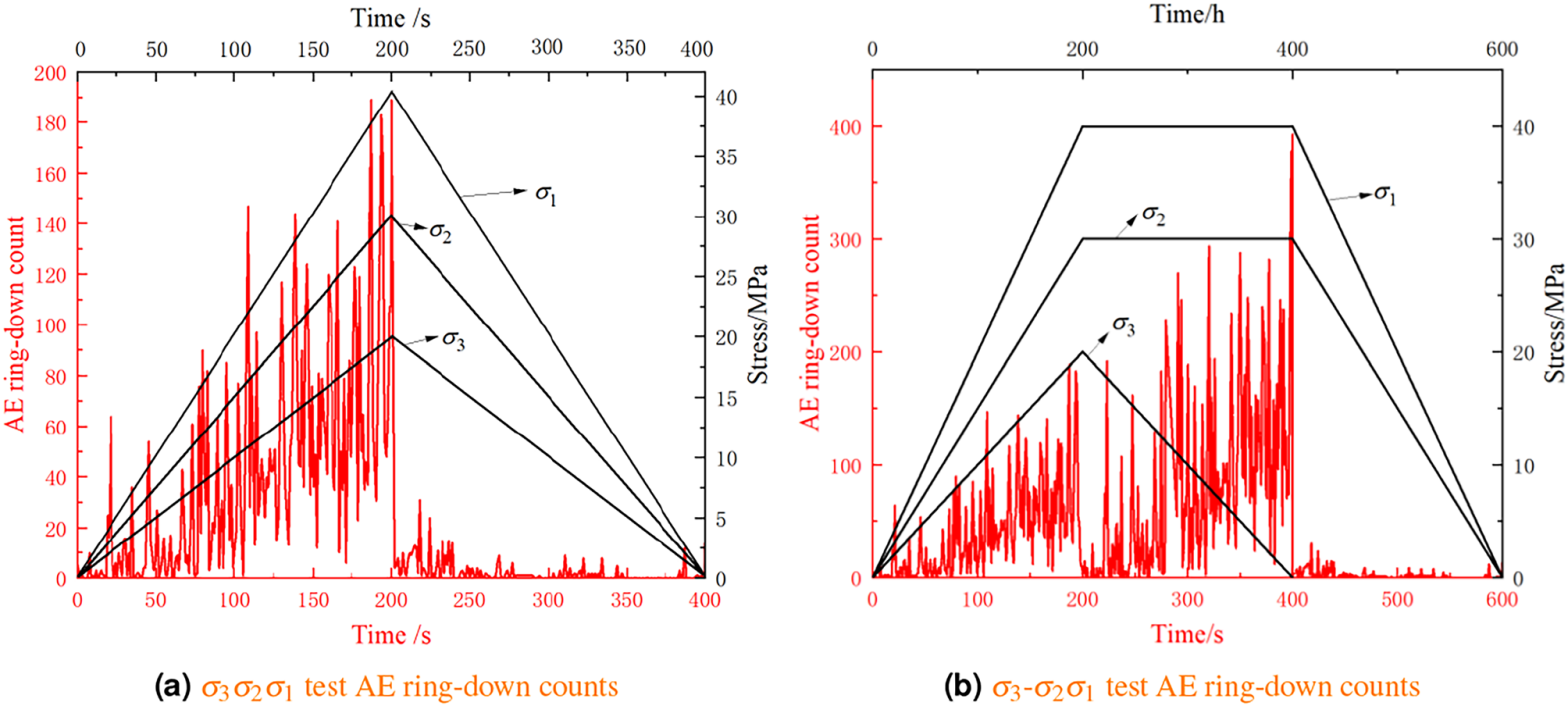
$\sigma _{3}$ direction is unloaded, there is a rise in SE density.

In the true triaxial stress equilibrium unloading test, the three principal stress directions are unloaded simultaneously and reduced to zero simultaneously. Theoretically, the area enclosed by the unloading stress-strain curve and the strain axis in the three principal stress directions represents the density of elastic SE released in the three principal stress directions. Based on the above research findings on AE, AE tests were conducted on the true triaxial stress equilibrium unloading test to observe the intensity of rock AE ring-down counts during the stress proportional reduction stage, thereby determining whether damage occurs. Based on the above analysis, AE ring-down counts was conducted on the true triaxial stress equilibrium unloading test, and the test results are shown in Fig. 10.

From Fig. 10, it can be seen that during the true triaxial loading stage, the intensity of AE ring-down counts in rock gradually increases with the stress level. In the $\sigma _{3}$-$\sigma _{2}$ $\sigma _{1}$ experiment, the intensity of acoustic emissions during the unloading of $\sigma _{3}$ is higher than during true triaxial loading. This indicates that under non-equilibrium stress unloading conditions, there is crack initiation and propagation within the rock. In both the $\sigma _{3}$ $\sigma _{2}$ $\sigma _{1}$ and $\sigma _{3}$-$\sigma _{2}$ $\sigma _{1}$ experiments, AE ring-down counts enter a quiet period after entering the stress equilibrium unloading stage, indicating that no new cracks are formed inside the rock. In summary, it can be concluded that in the stress equilibrium unloading test proposed in this paper, no damage occurs in the rock. That is, the unloading process in the true triaxial stress equilibrium unloading test is a process of elastic SE density release, validating the correctness of the SE density calculation method proposed in this paper.

## SE density analysis of true triaxial unloading principal stress test

According to thermodynamics, the failure of rocks is viewed as an imbalance phenomenon fueled by energy^1^. Analyzing the rock failure process from an energy perspective, rather than a stress-strain perspective, aids in understanding the nature of rock failure.

### $\sigma _{3} -\sigma _{2} -\sigma _{1}$ test SE density analysis

Based on the SE density calculation method proposed for the true triaxial unloading principal stress test, the SE density during the unloading principal stress test is calculated.

**Figure 11:**
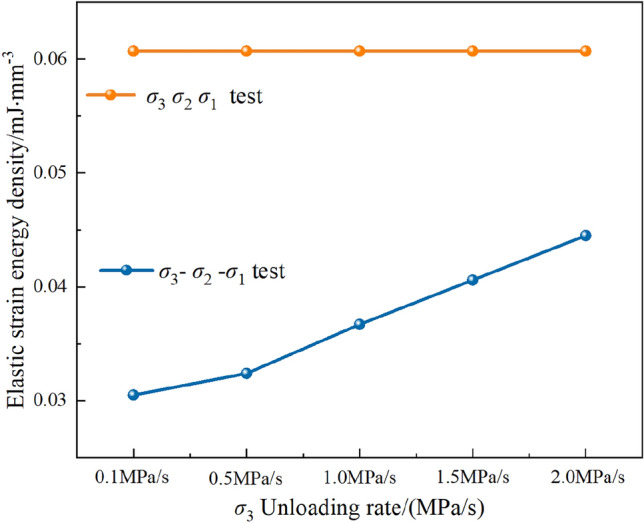
SE density of $\sigma _{3}$.

According to Fig. 11, the elastic SE released after unloading the principal stress during the $\sigma _{3}\sigma _{2}\sigma _{1}$ experiment is essentially equal under difference rate, suggesting that the unloading rate does not impact the density of the elastic SE released. Compared with the elastic SE density stored in the $\sigma _{3}$ direction under the initial stress level, the density of the elastic SE released by unloading the principal stress in the $\sigma _{3}$ direction during the $\sigma _{3} -\sigma _{2} -\sigma _{1}$ experiment is significantly reduced, indicating that the unloading stress path under true triaxial compression exerts a major influence on the release of elastic SE in sandstone. Additionally, the unloading rates also affect the elastic SE density in this direction. Higher unloading rates result in a greater density of elastic SE released in this direction, while a lower density of elastic SE is converted into dissipated SE during unloading.

**Figure 12:**
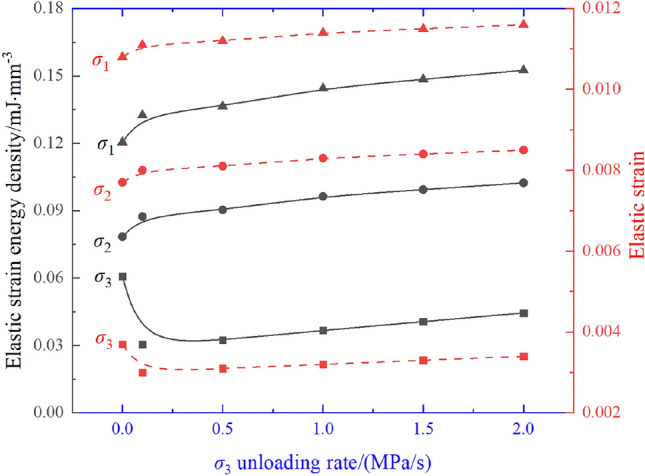
Elastic SE density and elastic strain at different unloading rates.

Under true triaxial compression conditions, the primary concern is the influence of initially unloading $\sigma _{3}$ on the accumulation and dissipation process of SE density in the other two principal stress directions ($\sigma _{2}$ and $\sigma _{1}$) is the core issue. To further explore this phenomenon, the unloading rate of $\sigma _{3}$ in the $\sigma _{3}\sigma _{2}\sigma _{1}$ experiment was set to 0, enabling a clearly compare and analyze the change pattern of elastic SE density in the $\sigma _{2}$ and $\sigma _{1}$ directions after unloading $\sigma _{3}$. The detailed evolution pattern is shown in Fig. 12.

From the data comparison in Fig. 12 shows that during the $\sigma _{3} -\sigma _{2} -\sigma _{1}$ experiment, the unloading of the $\sigma _{3}$ results in a significant variation in the released elastic SE density along the $\sigma _{3}$ within the sandstone. As the unloading rate gradually increases, the released elastic SE density in this direction tends to rise. However, overall, the released elastic SE density remains less than the stored elastic SE density along that direction. This indicates that the elastic SE density dissipates or transfers in this direction during the unloading of $\sigma _{3}$. The elastic strain associated with unloading $\sigma _{3}$ shows the same evolution trend as the elastic SE density in that direction. The increase in elastic strain implies a reduction in residual strain in the direction of $\sigma _{3}$, indicating that as the unloading rate of the $\sigma _{3}$ increases, the residual strain in that direction decreases. Simultaneously, an increase in the elastic SE density in the $\sigma _{2}$ and $\sigma _{1}$ directions was observed, indicating that unloading $\sigma _{3}$ not only affected that particular direction but also promoted the accumulation of SE in the remaining two principal stress directions. Furthermore, it is worth noting that the observed increase in elastic SE density in the $\sigma _{2}$ and $\sigma _{1}$ directions during the $\sigma _{3} -\sigma _{2} -\sigma _{1}$ experiment was actually significantly effect by the unloading rate of $\sigma _{3}$. As the unloading rate increased, the elastic SE density in the $\sigma _{2}$ and $\sigma _{1}$ directions also showed an increasing trend. Under uniaxial compression conditions, the unloading process primarily results in the release of elastic SE in a single direction. However, under true triaxial compression conditions, the situation is much more complex. To sum up, the process of principal stress unloading under true triaxial compression is more complex, covering many aspects such as energy storage, dissipation, transformation and release.

**Figure 13:**
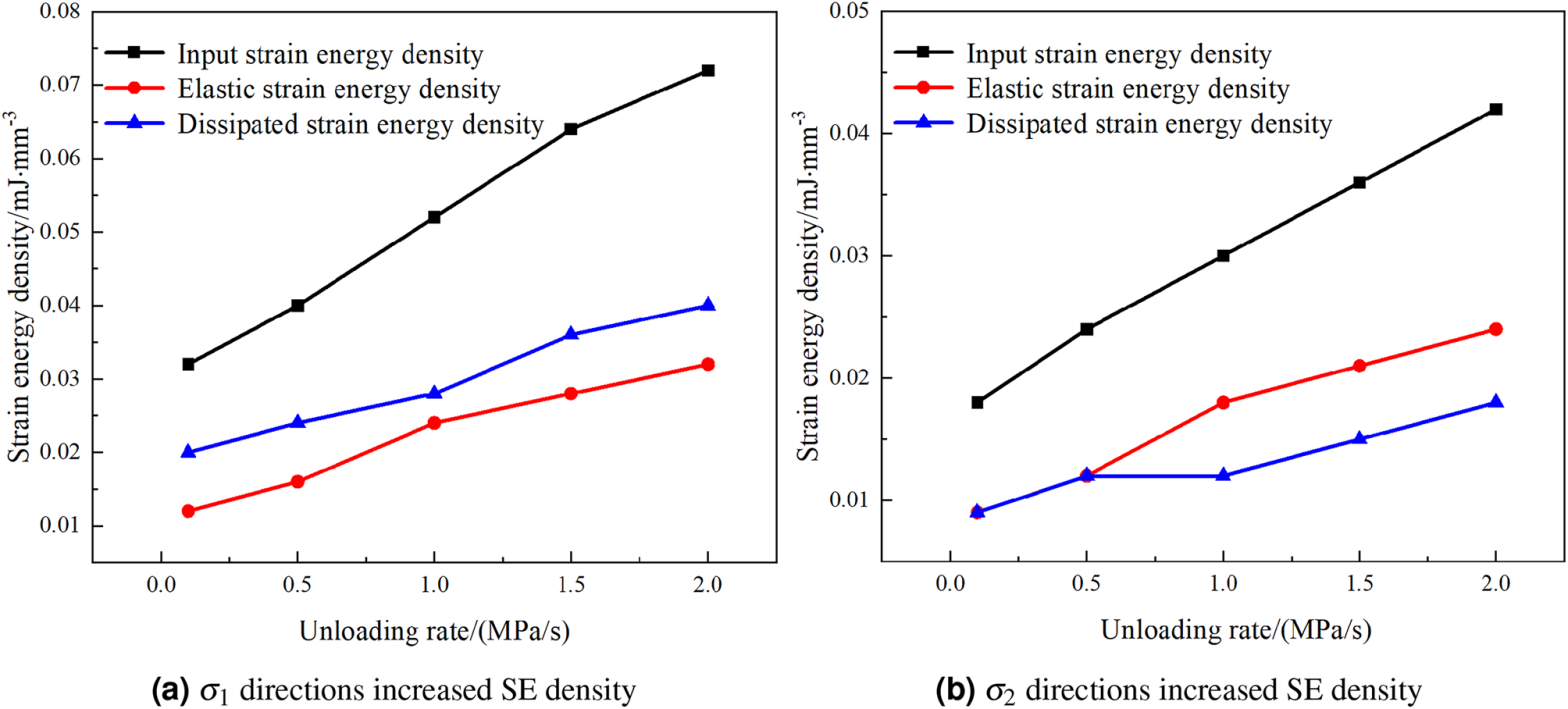
$\sigma _{3}$ direction is unloaded, there is a rise in SE density.

From the analysis of Fig. 13, it can be observed that after the main stress in the $\sigma _{3}$ direction is unloaded, there is a rise in SE density in both the $\sigma _{2}$ and $\sigma _{1}$ directions.

This rise in SE is not unidimensional but can be further categorized into two types: elastic SE density and dissipative SE density. Specifically, by comparing the detailed data and images of Fig. 13a and b, we find that under true triaxial compression of rocks, when the $\sigma _{3}$ is unloaded, the input SE density–whether it be elastic SE density or dissipative SE density–in both $\sigma _{2}$ and $\sigma _{1}$ directions increases with the rate of unloading.

**Figure 14:**
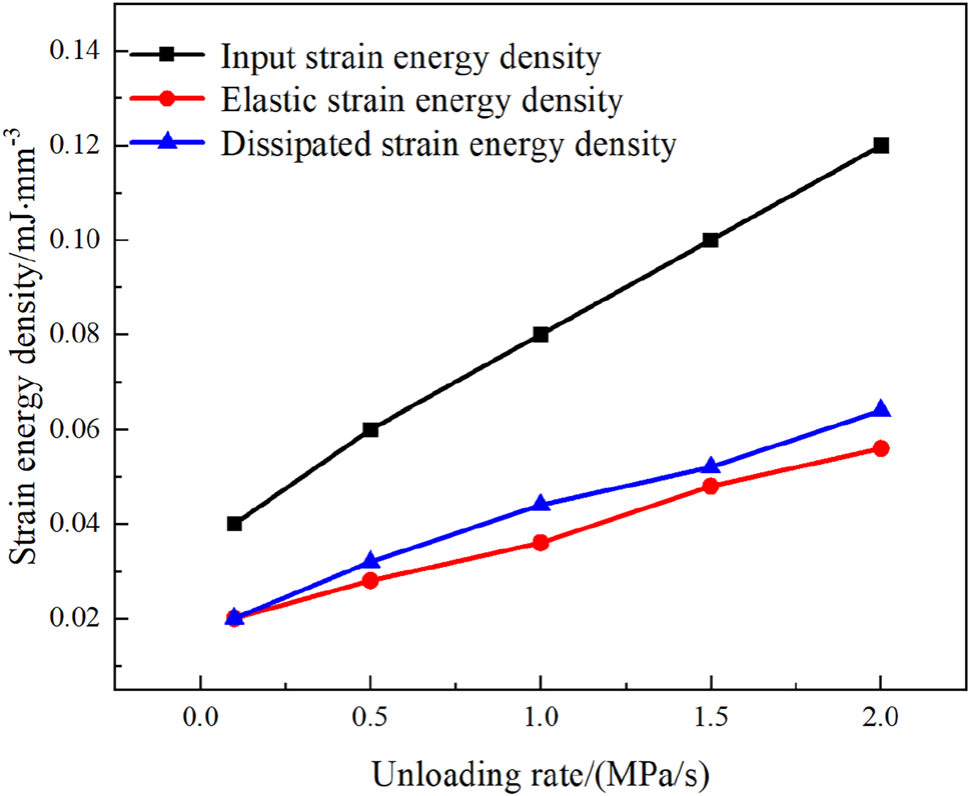
$\sigma _{2}$ direction is unloaded, rise in SE density of $\sigma _{1}$.

Further analysis shows that after the $\sigma _{3}$ direction stress is unloaded to 0, the unloading of the main stress in the $\sigma _{2}$ direction also induces an increase in SE density in the $\sigma _{1}$ direction, as illustrated in Fig. 14.

Some scholars studying the evolution of rock fractures under complex stress conditions believe that changes in the rock’s principal stress can induce the redistribution and expansion of rock fractures, changes in fracture patterns, and the closing of existing fractures and activation of microfractures^44–50^. During the process of spatial and shape changes of fractures, energy dissipation often accompanies, and Meng Qingbin et al. suggest that friction between rock fractures causes dissipation of internal SE^51^.

**Figure 15:**
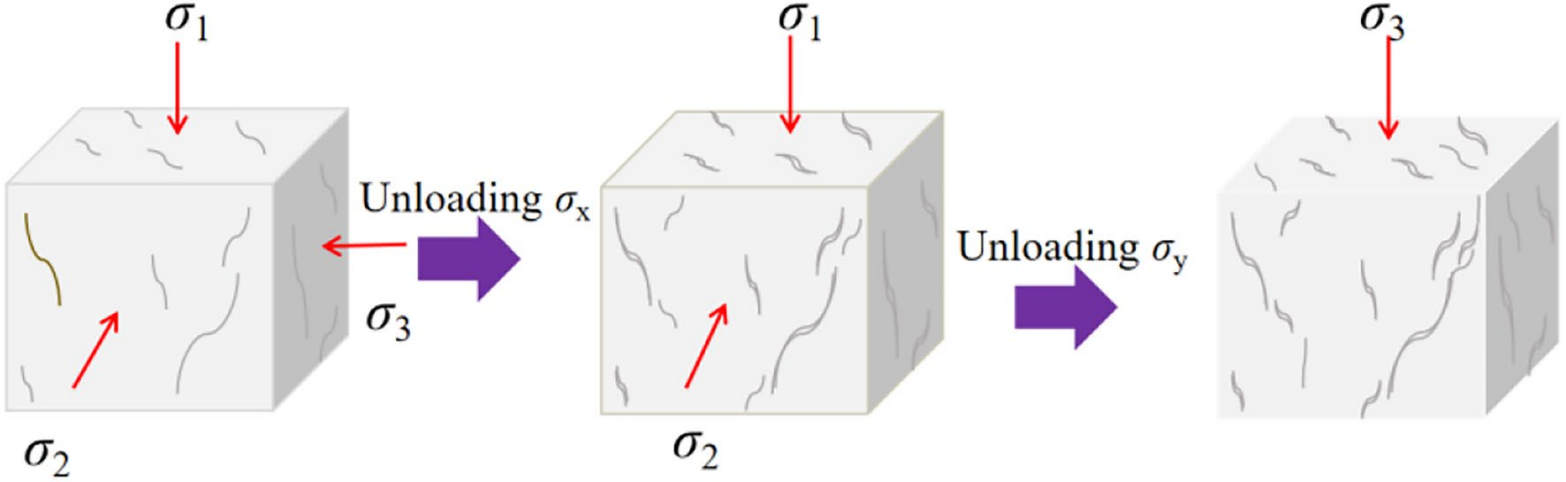
The crack evolution diagram.

More specifically, when there is a change in the principal stress within a rock, existing fractures may close due to stress redistribution, while new fractures may form, and existing ones may further propagate. The evolution of these fractures involves not only adjustments in their shape and orientation but also changes in their interstitial spaces. During the process of fracture morphological changes and spatial reorganization, friction lead to energy dissipation. The crack evolution diagram is shown in Fig. 15.

Experimental results indicate that the rate of unloading significantly affects this process. A faster unloading rate gives internal fractures in rocks less time to adapt to the new stress state, leading to higher stress concentration and consequently more energy dissipation and storage during this process. This explains why a higher unloading rate in one principal stress direction results in greater SE density accumulation and dissipation in the other two principal stress directions. Therefore, an increase in the unloading rate leads to an increase in both input SE density and dissipated SE density in other principal stress directions.

### Energy dissipation is associated with damage

**Figure 16:**
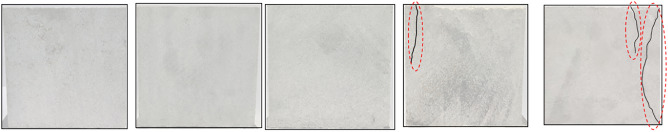
$\sigma _{3}-\sigma _{2}-\sigma _{1}$ test unloading. (The unloading rate is 0.1MPa/s, 0.5MPa/s, 1.0MPa/s, 1.5MPa/s, and 2.0MPa/s from left to right.).

As shown in Fig. 16, observing the morphology of specimens after unloading in the $\sigma _{3}-\sigma _{2}-\sigma _{1}$ test, test reveals that at unloading rates of 0.1, 0.5, and 1.0MPa/s, no significant crack formations were detected on the rock surface, indicating the sandstone structure remains relatively intact with minor damage at these rates. However, when the unloading rate increased to 1.5MPa/s, noticeable crack formation began to appear, indicating the initial damage to the rock. Moreover, when the unloading rate was increased to 2.0MPa/s, the number and size of the surface cracks on the rock were significantly greater than those at 1.5MPa/s, indicating more severe damage.

Detailed analysis of SE density in this study shows a clear trend: as the unloading rate increases, stress concentration inside the rock becomes more significant, leading to more internal friction between cracks, and consequently, a greater density of dissipated SE. Many scholars agree on the proportional relationship between dissipative SE density and the extent of rock damage. Combined with this paper’s experimental results, it can be concluded that an increase in the unloading rate leads to progressively more severe rock damage following the unloading process under true triaxial conditions.

## Conclusions

In order to investigate how the unloading of principal stresses impacts the change in SE density within rock masses under true triaxial compression, this research designed a stress balance unloading experiment and carried out a detailed analysis of the SE density for the $\sigma _{3}-\sigma _{2}-\sigma _{1}$ test. The primary findings of this research are as follows:

1. Unloading any principal stress in true triaxial conditions typically elevates SE density in remaining directions, categorized into reversible elastic and irreversible dissipative SE densities.
2. The elastic SE density released during unloading is influenced by the stress path and rate, being typically lower than that stored during true triaxial compression. Lower unloading rates result in minimal elastic SE release and greater conversion to dissipated SE.
3. Faster unloading speeds delay stress adjustments, increasing stress concentration and consequently amplifying the SE density, both elastic and dissipative, in the other principal stress directions.
4. Unloading the principal stress induces a positive correlation between the dissipated SE generated in the other principal stress directions and rock damage, manifested as an increase in the formation of cracks after unloading with an increase in the unloading rate.
5. A novel calculation method for SE density in true triaxial unloading tests is introduced, enriching the analysis of SE density variations under complex stress conditions.

## Data Availability

Data sets generated and/or analyzed during the current study period may have an impact on the subsequent development of the study due to the disclosure of preliminary data but may be obtained from the corresponding author upon reasonable request.

## References

[1] Xie H, Lu J, Li C, Li M, Gao M (2022). Experimental study on the mechanical and failure behaviors of deep rock subjected to true triaxial stress: A review. Int. J. Min. Sci. Technol..

[2] Peng R (2015). Energy dissipation and release during coal failure under conventional triaxial compression. Rock Mech. Rock Eng..

[3] He M, Huang B, Zhu C, Chen Y, Li N (2018). Energy dissipation-based method for fatigue life prediction of rock salt. Rock Mech. Rock Eng..

[4] Wang C (2020). Stress-energy mechanism for rock failure evolution based on damage mechanics in hard rock. Rock Mech. Rock Eng..

[5] Richards Melissa C, Issen Kathleen A, Ingraham Mathew D (2022). A coupled elastic constitutive model for high porosity sandstone. Int. J. Rock Mech. Min. Sci..

[6] Thongprapha T, Tengpakwaen K, Daemen J, Fuenkajorn K (2022). Effect of confining pressures on transverse isotropy of Maha Sarakham salt. Int. J. Rock Mech. Min. Sci..

[7] Li C, Gao C, Xie H, Li N (2020). Experimental investigation of anisotropic fatigue characteristics of shale under uniaxial cyclic loading. Int. J. Rock Mech. Min. Sci..

[8] Yang S-Q, Yin P-F, Li B, Yang D-S (2020). Behavior of transversely isotropic shale observed in triaxial tests and brazilian disc tests. Int. J. Rock Mech. Min. Sci..

[9] Li M (2014). Effect of specimen size on energy dissipation characteristics of red sandstone under high strain rate. Int. J. Min. Sci. Technol..

[10] Jiabing Z, Ronghuan D, Yiling C, Zhen H (2023). Experimental investigation of the mechanical properties and energy evolution of layered phyllite under uniaxial multilevel cyclic loading. Rock Mech. Rock Eng..

[11] Gao Y, Feng X-T (2019). Study on damage evolution of intact and jointed marble subjected to cyclic true triaxial loading. Eng. Fract. Mech..

[12] Hongxin X, Xuehua L, Changhao S, Ze X, Liqiang Y (2023). Study on the damage mechanism and energy evolution characteristics of water-bearing coal samples under cyclic loading. Rock Mech. Rock Eng..

[13] Liu X, Ning J, Tan Y, Gu Q (2016). Damage constitutive model based on energy dissipation for intact rock subjected to cyclic loading. Int. J. Rock Mech. Min. Sci..

[14] Wang Y, Feng WK, Hu RL, Li CH (2021). Fracture evolution and energy characteristics during marble failure under triaxial fatigue cyclic and confining pressure unloading (fc-cpu) conditions. Rock Mech. Rock Eng..

[15] Jianguo N (2018). Estimation of crack initiation and propagation thresholds of confined brittle coal specimens based on energy dissipation theory. Rock Mech. Rock Eng..

[16] Zhao K (2020). Energy evolution of brittle granite under different loading rates. Int. J. Rock Mech. Min. Sci..

[17] Fengqiang G, Jingyi Y, Xibing L (2018). A new criterion of rock burst proneness based on the linear energy storage law and the residual elastic energy index. Chin. J. Rock Mech. Eng..

[18] Gong F, Yan J, Luo S, Li X (2019). Investigation on the linear energy storage and dissipation laws of rock materials under uniaxial compression. Rock Mech. Rock Eng..

[19] Gong F, Yan J, Li X, Luo S (2019). A peak-strength strain energy storage index for rock burst proneness of rock materials. Int. J. Rock Mech. Min. Sci..

[20] Peng K (2021). Quantitative characteristics of energy evolution of gas-bearing coal under cyclic loading and its action mechanisms on coal and gas outburst. Rock Mech. Rock Eng..

[21] Zhang Z (2019). Deformation damage and energy evolution characteristics of coal at different depths. Rock Mech. Rock Eng..

[22] Wang J, Song W, Cao S, Tan Y (2019). Mechanical properties and failure modes of stratified backfill under triaxial cyclic loading and unloading. Int. J. Min. Sci. Technol..

[23] Shuang D (2023). Strain evolution and fatigue damage characteristics analysis of sandstones during multi-level triaxial cyclic loading and unloading under varying stress limits. Rock Mech. Rock Eng..

[24] Iwata N, Sasaki T, Yoshinaka R, Kurooka K (2012). Applicability of the multiple yield model for estimating the deformation of vertical rock walls during large-scale excavations. Int. J. Rock Mech. Min. Sci..

[25] Feng GA, Zhou KP, Luo XW, Zhai JB (2012). Effect of induction unloading on weakening of rock mechanics properties. Trans. Nonferrous Metals Soc. China.

[26] Manchao H, Fei Z (2013). Laboratory study of unloading rate effects on rockburst. Disaster Adv..

[27] Huang D, Li Y (2014). Conversion of strain energy in triaxial unloading tests on marble. Int. J. Rock Mech. Min. Sci..

[28] Li Y, Han L, Shang T (2024). Effects of complex triaxial unloading confining stress conditions on the mechanical behaviour and fracture mechanism of sandstone. Int. J. Min. Sci. Technol..

[29] Yunfei W, Liping W, Huazhe J, Hongbo Z (2015). Mechanical charactristics of deformation and damage mechanism of sandstone under different confining pressure. Coal Geol. Explor..

[30] Su G, Zhai S, Jiang J, Zhang G, Yan L (2017). Influence of radial stress gradient on strainbursts: An experimental study. Rock Mech. Rock Eng..

[31] Martin CD, Chandler NA (1994). The progressive fracture of Lac du Bonnet granite. Int. J. Rock Mech. Min. Sci. Geomech. Abstr..

[32] Eberhardt E, Stead D, Stimpson B, Read RS (1998). Identifying crack initiation and propagation thresholds in brittle rock. Can. Geotech. J..

[33] Diederichs MS, Kaiser PK, Eberhardt E (2004). Damage initiation and propagation in hard rock during tunnelling and the influence of near-face stress rotation. Int. J. Rock Mech. Min. Sci..

[34] Jiang Quan, Feng Xia-ting, Fan Yilin, Fan Qixiang, Liu Guofeng, Pei Shufeng, Duan Shuqian (2017). In situ experimental investigation of basalt spalling in a large underground powerhouse cavern. Tunn. Undergr. Space Technol..

[35] Zhao JS, Zhao YM, Li PX, Chen CF, Zhang JC, Chen JH (2023). Microseismic monitoring of the fracture nucleation mechanism and early warning for cavern rock masses. Processes.

[36] Tianbin Li, Lansheng Wang (1993). An experimental study on the deformation and failure features of a basalt under unloading condition. Chin. J. Rock Mech. Eng..

[37] Li H, Xia C, Yan Z, Jiang K, Yang L (2007). Study on marble unloading mechanical properties of Jinping hydropower station under high geostress conditions. Yanshilixue Yu Gongcheng Xuebao/Chin. J. Rock Mech. Eng..

[38] Hua An-Zeng, You Ming-Qing (2001). Rock failure due to energy release during unloading and application to underground rock burst control. Tunn. Undergr. Space Technol..

[39] Li D, Sun Z, Xie T, Li X, Ranjith PG (2017). Energy evolution characteristics of hard rock during triaxial failure with different loading and unloading paths. Eng. Geol..

[40] Seleznev M, Weidner A, Biermann H (2024). Detection of early fatigue damage during ultrasonic fatigue testing of steel by acoustic emission monitoring. Int. J. Fatigue.

[41] Jingmang Xu, Wang Kai, Ma Qiantao, Li Haoran, Wang Ping, Chen Rong, Qian Yao, Zeng Dongfang (2023). Study on acoustic emission properties and crack growth rate identification of rail steels under different fatigue loading conditions. Int. J. Fatigue.

[42] Nor NM, Abdullah S, Saliah SM (2021). On the need to determine the acoustic emission trend for reinforced concrete beam fatigue damage. Int. J. Fatigue.

[43] Zhao Y, Gong S, Teng TE, Jiang Y, Yang Z, Chen K (2018). Characteristics of the load/unload response ratio of raw coal under uniaxial multi-level cyclic loading. Chin. J. Rock Mech. Eng..

[44] Zhao X, Zhou T, Zhai T, Ju Y, Zhu J (2023). Experimental investigation on crack initiation and damage stresses of deep granite under triaxial compression using acoustic methods. J. Rock Mech. Geotech. Eng..

[45] Rao Q, Zhao C, Yi W (2023). A new mixed-mode phase-field model for crack propagation of brittle rock. J. Rock Mech. Geotech. Eng..

[46] Zhang D, Zhu W, Li S, Zhang B, Wang W (2012). A modified maximum tangential tensile stress criterion for three-dimensional crack propagation. J. Rock Mech. Geotech. Eng..

[47] Chen LX, Guo WY, Zhang DX, Zhao TB (2022). Experimental study on the influence of prefabricated fissure size on the directional propagation law of rock type-i crack. Int. J. Rock Mech. Min. Sci..

[48] Chen Y, Zuo J, Li Z, Dou R (2020). Experimental investigation on the crack propagation behaviors of sandstone under different loading and unloading conditions. Int. J. Rock Mech. Min. Sci..

[49] Yang YX (2022). A virtual crack-based numerical manifold approach to crack initiation, propagation and coalescence in granite. Rock Mech. Rock Eng..

[50] Dai J, Liu J, Zhou L, He X (2023). Crack pattern recognition based on acoustic emission waveform features. Rock Mech. Rock Eng..

[51] Meng Q, Zhang M, Han L, Pu H, Nie T (2016). Effects of acoustic emission and energy evolution of rock specimens under the uniaxial cyclic loading and unloading compression. Rock Mech. Rock Eng..

